# Neuroleukemiosis Masquerading as Drug Toxicity in an Adolescent With Refractory AML

**DOI:** 10.1002/ajh.70006

**Published:** 2025-07-23

**Authors:** Nia Choi, Deborah Schady, Tim Lotze, Jill Ann Jarrell, Alexandra Rodriguez‐Hernandez, Sandra Aziz, Karen K. Moeller, Joanna S. Yi, Suzanne Woodbury, Andrea N. Marcogliese, Zoann E. Dreyer, Eric S. Schafer

**Affiliations:** ^1^ Department of Pediatrics Baylor College of Medicine Houston Texas USA; ^2^ Department of Pathology and Immunology Baylor College of Medicine Houston Texas USA; ^3^ Neuroscience Center Texas Children's Hospital Houston Texas USA; ^4^ Division of Palliative Care, Baylor College of Medicine Texas Children's Hospital Houston Texas USA; ^5^ Department of Radiology Texas Children's Hospital Houston Texas USA; ^6^ Texas Children's Cancer and Hematology Center Texas Children's Hospital Houston Texas USA; ^7^ Department of Physical Medicine and Rehabilitation Baylor College of Medicine Houston Texas USA

**Keywords:** AML, *KMT2a*‐rearranged, menin‐inhibitor, neuroleukemiosis

## 1 Case Presentation

**A 15‐year‐old male presented with the acute onset of fever, fatigue, headaches, hyperleukocytosis, anemia, and thrombocytopenia and was ultimately diagnosed with a t(9;11) [KMT2A::MLLT3] KMT2A‐rearranged (KMT2A‐r) acute myeloid leukemia (AML). Head computed tomography (CT) showed a 4 mm intracranial lesion presumed to represent hemorrhage, and diagnostic lumbar puncture (LP) noted no evidence of leukemia in the cerebrospinal fluid (CSF). The patient started therapy, during which he achieved a minimal residual disease (MRD) negative complete remission at the end of Induction I**.

The patient and family were offered therapy on a Children's Oncology Group clinical trial (AAML1831, NCT04293562), but they chose to be treated on the local best practice standard, which was based largely on the standard arm of that study. The patient's *KMT2A::MLLT3* fusion and post‐Induction 1 (Cycle 1) MRD negative response placed him into a “Low Risk 2” category for which he would subsequently receive four additional 28‐day cycles of chemotherapy termed Induction 2, Intensification 1, Intensification 2, and Intensification 3 without hematopoietic stem cell transplant providing an estimated 5‐year disease free survival of 63.7% ± 4.5% [1].

**After recovery from Induction I, the patient received Induction II therapy with minimal complications; however, before Intensification I, the patient presented with new left‐sided facial weakness. Brain magnetic resonance imaging (MRI) showed multifocal lesions throughout the supratentorial white matter. A biopsy of the area revealed myeloid sarcoma. High‐dose cytarabine (HD‐AraC) with asparaginase (“Capizzi AraC”) was initiated for relapsed disease, during which extremity pain and global weakness developed, suggestive of a multifocal mixed motor and sensory neuropathy. The patient was started on acetaminophen, morphine, and gabapentin with steady improvement in pain. Physical and occupational therapy (PT and OT) enabled the patient to comfortably ambulate laps with a platform walker. Post‐therapy, bone marrow (BM) MRD and CSF were both negative for leukemia, and a brain MRI showed interval improvements of intracranial lesions**.

The patient experienced a surprising, early, on‐therapy, isolated central nervous system (CNS) relapse of his AML. Post‐infusion asparaginase potentiates the antileukemic effect of HD‐AraC and is effective in treating CNS disease [2]; and so, this “Capizzi II” regimen is commonly used in both de novo (often as part of Intensification therapy) [3] and relapsed AML [2] regimens. However, CNS toxicities are well described and include headache, seizure, and somnolence [4]. Cytarabine‐induced peripheral neuropathies have also been reported [4, 5, 6]. While the etiology of his neuropathies was unconfirmed, it was presumed to be drug exposure [7] versus chronic illness [8].

**Shortly after the disease reassessment, the patient experienced increased extremity pain, urinary incontinence and right arm weakness. A brain MRI showed increased size of previously noted intraparenchymal enhancing lesions. Spine MRI was negative for abnormalities. He received emergent cranial radiation therapy (CRT); within days of this, he experienced a seizure and mental status changes. Follow‐up OT/PT evaluations revealed minimal distal movement observed in wrists and digits with intrinsic muscle wasting and the lack of the ability to ambulate, although it was unclear if this was secondary to deconditioning, weakness, pain, or a combination thereof**.

Given the rapid onset of new CNS symptoms in such proximity to excellent CNS‐penetrating chemotherapy, management with CRT was chosen. The Pediatric Advanced Care Team (PACT), the hospital's palliative care service, was consulted for refractory neuropathic pain, patient and family coping, assistance with elucidating goals of care, and complex medical decision‐making [9]. Methadone was recommended due to its reported activity on μ‐opioid and *N*‐methyl‐d‐aspartate receptors, leading to efficacy in the treatment of both nociceptive and neuropathic types of pain [10]. Duloxetine, ketamine, topical analgesics, and non‐pharmacologic modalities were all trialed without significant improvement in pain.

**Neurology and Physical Medicine and Rehabilitation consultants recommended an electromyography (EMG) and the Invitae Comprehensive Neuropathies Panel (invitae.com/us/providers/test‐catalog/test‐03200) to further evaluate his advancing neurologic symptoms, but the family declined. Two weeks after radiation, recurrent/refractory (R/R) AML was noted in his BM, on brain MRI, and on fludeoxyglucose‐18 positron emission tomography (FDG‐PET) scans (which showed innumerable areas of enhancement throughout his entire body). The recurrent AML was refractory to new therapy with venetoclax and azacytidine (VEN‐AZA); therefore, the patient was started on a regimen of decitabine and vorinostat with fludarabine, cytarabine and filgrastim (DV‐FLAG) and twice weekly LPs with intrathecal chemotherapy (IT triples [cytarabine, hydrocortisone, and methotrexate] alternating with IT doubles [cytarabine and hydrocortisone])**.

*KMT2A*‐r leukemias are known to have dysregulated transcription secondary to hypermethylated gene signatures, which can be restored by epigenetic agents such as DNA methyltransferase inhibitors (DNMTis) (e.g., azacytidine [AZA] and decitabine [11]) and histone deacetylase inhibitors (HDACis) (e.g., vorinostat). Based on most AMLs (including *KMT2a*‐r) having high BCL‐2 expression, VEN‐AZA is now considered standard of care in the treatment of R/R AML in younger adults and in de novo AML in older adults who are not fit for intensive therapy [12]. When our patient's disease proved to be resistant to this therapy, we advanced to the more intensive DV‐FLAG, another epigenetic therapy, now combined with cytotoxic therapy, which has been shown to be effective in pediatric patients with R/R *KMT2A*‐r AML [13].

We also continued to pursue an etiology of his unusual, treatment‐resistant, and progressive neuropathy. Nerve conduction studies (NCS) and EMG are required to discern between demyelinating and axonal nerve injury. Whereas demyelinating diseases of the peripheral nerves (PN) produce a slowing of the nerve action potential conduction velocity, axonal diseases cause a decreased amplitude of the action potential. These studies may additionally be able to distinguish between hereditary—which have more uniform (and often more severe) slowing—and acquired diseases—which generally produce uneven degrees of myelin loss of different motor and sensory nerves, resulting in varying degrees of conduction slowing [14].

**FIGURE 1:**
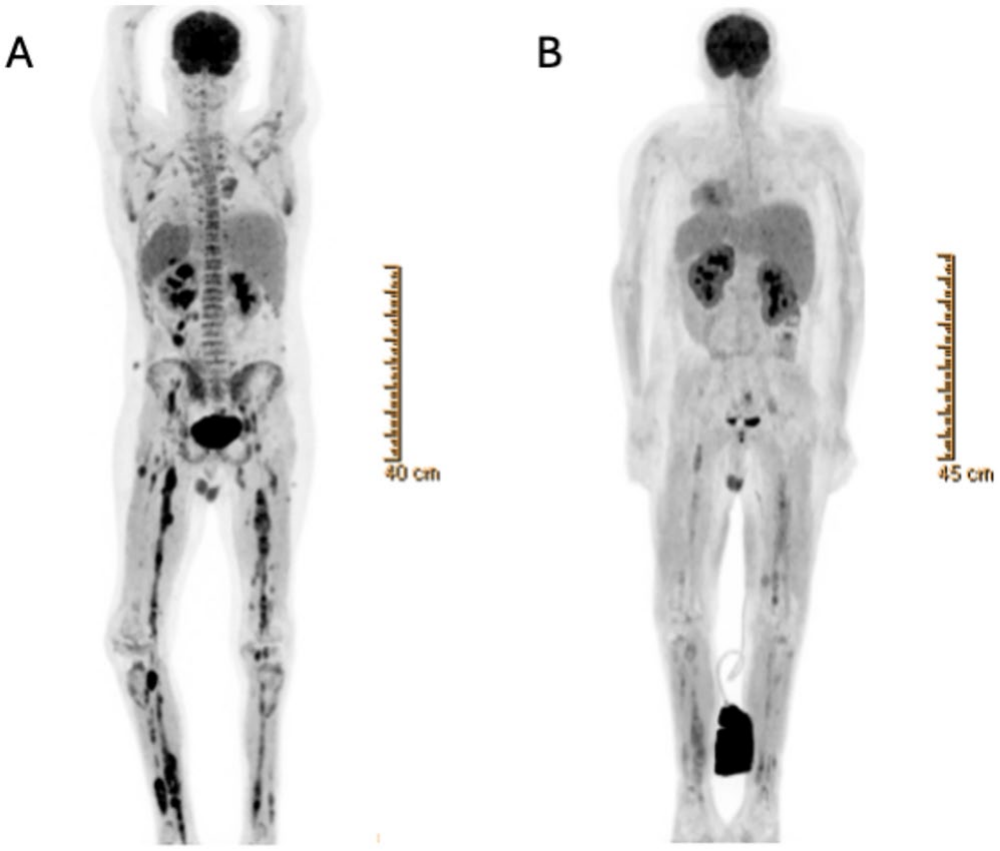
(A) FDG‐PET scan before initiation of revumenib showing extensive uptake representing active AML in all extremities lower > upper, neck and trunk. (B) FDG‐PET scan after 1 month of revumenib showing dramatic decreased uptake throughout. Examples of standardized uptake value (SUV) improvements from A (SUV_A_) to B (SUV_B_) include: Gastrostomy tract (SUV_A_: 10.8, SUV_B_: 4.7); splenic tail (SUV_A_: 6, SUV_B_: Signal intensity no longer above background); right anterior abdominal wall lesion (SUV_A_: 7.9, SUV_B_: 0.7) and left external iliac node (SUV_A_: 6.4, SUV_B_: 2.7). [Color figure can be viewed at wileyonlinelibrary.com]

**At his nadir of DV‐FLAG‐induced myelosuppression, the patient developed viral septic shock, after which he was noted to have complete stocking‐glove neuropathy with loss of deep tendon reflexes and 0/5 strength at the wrists, ankles, and interosseous muscles bilaterally. Neuropathy‐directed testing was again recommended, and this time it was performed. EMG demonstrated severe axonal sensory‐motor polyneuropathy below the elbow and below the knee muscles with denervation. AML re‐evaluation noted a BM with 13.5% leukemic blasts, CSF with the presence of leukemic blasts, FDG‐PET scan with progressive disease signal throughout the extremities, neck, chest, abdomen, and pelvis** (Figure 1A), **and brain/spine MRI showed stable but persistent disease. Then, it was concluded that there were no additional logical, commercially available AML‐directed options to pursue**.

The patient's electrodiagnostic testing showed severe axonal sensory‐motor neuropathy with all the tested sensory and motor nerves having absent responses, and EMG showing denervation potentials in the upper‐proximal and lower‐distal extremity muscles tested. The differential diagnosis for axonal sensory‐motor polyneuropathies is wide, but commonly includes inflammatory neuropathies such as axonal variant Guillain–Barré syndrome (GBS), toxic neuropathies (common with chemotherapy agents), hereditary neuropathies (potentially subclinical and unmasked by exposure to toxic chemotherapeutic medications), metabolic neuropathies, nutritional disturbances, infection, connective tissue disorders, and other systemic illness [14]. Given the possibility of a toxic and hereditary dual pathology, genetic testing for pathogenic variants in neuropathy‐related genes was important and was finally collected.

Despite multimodal analgesia, the patient's pain worsened. PACT worked to address his total pain (the suffering that encompasses all of a person's physical, psychological, social, spiritual, and practical struggles [15]) by augmenting pharmacological interventions with strategies targeting the patient's nonphysical aspects of pain, such as mood and psychosocial stressors. PACT attempted to elucidate the patient's goals of care and facilitate shared decision‐making between the patient, family, and all care teams in the setting of an extremely poor prognosis.

**The patient and family chose to continue disease‐directed therapy. Eight months from the initial diagnosis of AML, the patient was started on single‐agent revumenib (SNDX‐5613), a novel, oral menin inhibitor, under an expanded access program (EAP, NCT05918913). Weekly triple IT therapy was continued. A few days after revumenib initiation, the patient experienced differentiation syndrome requiring the temporary initiation of hydroxyurea, dexamethasone, and aggressive diuresis for fluid overload** (https://cms.syndax.com/wp‐content/uploads/Revuforj‐full‐prescribing‐info.pdf). **However, within 2 weeks, the patient experienced a significant decrease in pain, allowing weaning of analgesia. After the first cycle (28 days) of revumenib, a disease re‐evaluation showed an MRD‐negative BM, clear CSF on cytology, stable lesions on brain MRI, and near resolution of all systemic avid lesions by FDG‐PET** (Figure 1B).

*KMT2A*‐rearrangements lead to oncogenic fusion proteins, which upregulate leukemogenic homeobox (*HOX*) genes that require the scaffolding protein menin for promoter binding [16]. Revumenib (SNDX‐5613) is a potent, oral, small‐molecule inhibitor of the menin–KMT2A complex, which in single‐agent early Phase 1 and 2 trials [17, 18] showed impressive safety and efficacy in patients with R/R *KMT2A*‐r leukemias. The overall response rate in the Phase 2 AUGMENT‐101 study was 63.2%, which supported its recent FDA approval (https://cms.syndax.com/wp‐content/uploads/Revuforj‐full‐prescribing‐info.pdf) for patients > 1 year with R/R acute leukemia and a *KMT2A* translocation. MRD negativity in patients with at least a partial hematologic recovery at the time of evaluation occurred in 7 of 57 (12%) of patients [18]. The main ≥ grade 3 adverse effects noted were prolonged QTc (13.8%)—reversible with dose reductions—and differentiation syndrome (16%)—successfully managed with corticosteroids and hydroxyurea. Based on preclinical studies, peripheral neuropathy was considered an adverse event of special interest but occurred in only 3.2% of patients [18]. Our patient demonstrated an impressive and rapid disease, pain, and quality of life response to revumenib. Interestingly, it was notable that he had not experienced any perceptible neurologic improvement.

**FIGURE 2:**
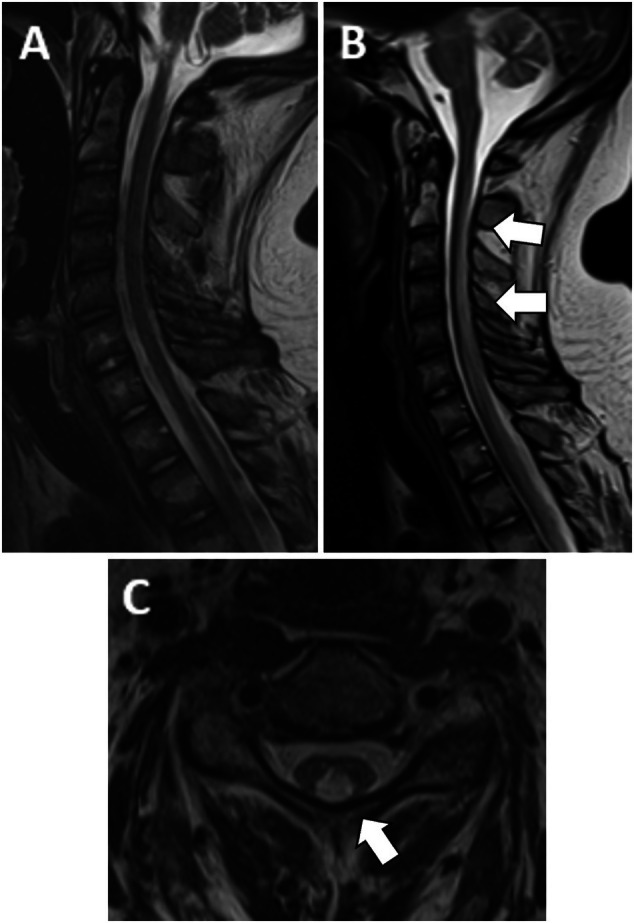
(A) Normal pre‐revumenib sagittal T2WI MRI of the cervical spine. (B) Sagittal T2WI MRI of the cervical spine 4 weeks later (post‐revumenib exposure) demonstrates abnormal signal (white arrows) in the posterior spinal cord. (C) Post‐revumenib axial T2WI MRI images confirm that the abnormality (white arrow) was confined to the posterior columns. This finding extended into the thoracic spinal cord (not shown).

**However, new findings on the brain/spine MRI showed new diffuse confluent white matter disease of the brain “consistent with methotrexate related leukoencephalopathy” and new holocord edema of the posterior columns with no evidence of cord compression or new paraspinal masses** (Figure 2). **Genetic testing with the Invitae Comprehensive Neuropathies panel returned negative results. Secondary to this radiologic finding, revumenib was emergently discontinued**.

Cord abnormalities that preferentially affect the posterior columns are most often a result of demyelination in the setting of vitamin B12 deficiency (subacute combined degeneration of the cord). Nitrous oxide can interfere with the B12 pathway, also leading to this pattern of demyelination. The differential diagnosis also includes vitamin E deficiency, neurosyphilis (tabes dorsalis) and while rare, several drugs/medications, including methotrexate [19], have been known to cause demyelination by either direct chemical‐induced neurotoxicity or indirectly by triggering an immune system dysregulation [20] An additional disease on the differential was GBS since it is an acquired demyelinating disease of the sensory and motor nerves. An acquired condition, like GBS, was considered in light of the negative genetic testing. However, it was thought to be an unlikely cause of our patient's new radiologic findings. The cord findings suggesting a demyelinating disease were categorically different from the axonal injury suggested by the patient's earlier PN EMG. As such, we were left to assume that the cord abnormalities were not a progression of his PN pathology, but rather a new phenomenon.

**FIGURE 3:**
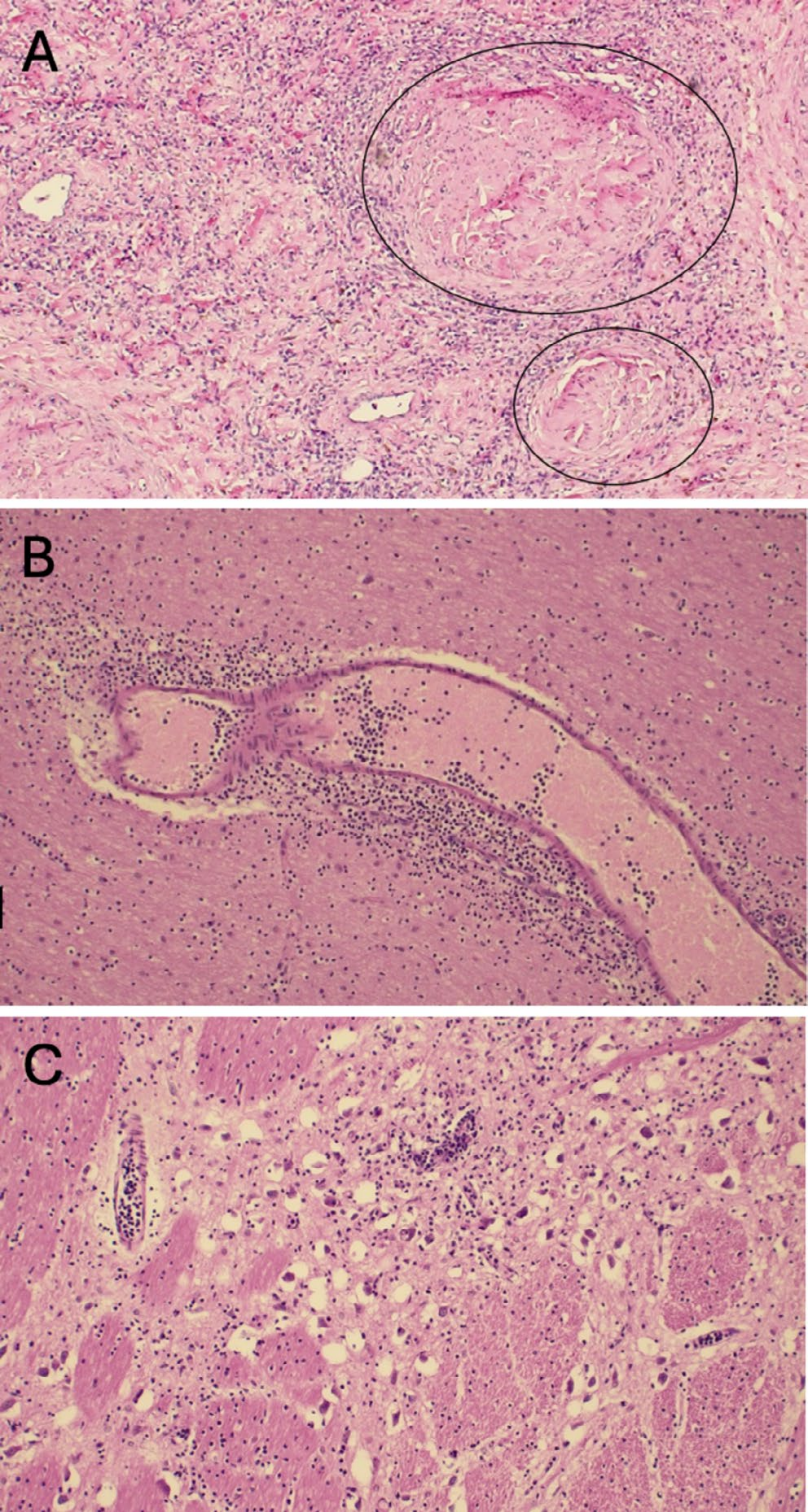
Autopsy specimens. (A) Cross‐section of sciatic nerve showing neurovascular bundles (circles) with extensive infiltration of the perineurium and endoneurium from clonal mononuclear cells of intermediate size and with large nuclei and scant cytoplasm (“deep purple cells”) representing the patient's AML. (B) Section of cerebral cortex with blood vessel in cross section; the vessel is surrounded by invading AML cells with extension into the deep glial tissue. (C) Section of pons with cross sections of blood vessels and neurons (clear circular structures). AML cells are seen in the blood vessels as well as in the neural tissue surrounding multiple neurons.

**Two weeks later, the patient was re‐challenged with revumenib at a 50% dose reduction** [18]. **Within 1 week of drug restart, the patient had new‐onset dysphagia. Ultrasound diagnosed hemiparesis of the vocal cords, and neck MRI revealed no anatomic explanation for this emergent neurologic finding. Given no other apparent etiology for this new neuropathy and the temporal relationship to drug re‐exposure, all parties agreed to permanently discontinue revumenib. Per the patient and family's wishes, the patient was transitioned to comfort‐focused care. Ten months after his initial AML diagnosis, the patient died from complications of disease progression. The family requested an autopsy, which revealed neuroleukemiosis of the PN without signs of demyelination** (Figure 3A). **There was leukemic infiltration seen in the CNS** (Figure 3B,C) **and the pituitary gland. The spinal cord was also diffusely necrotic. No evidence of demyelination was found in any area of the nervous system examined**.

## 2 Discussion

Our patient with multiply R/R *KMT2a*‐r AML demonstrated a remarkable near complete response to revumenib by all available conventional leukemia evaluation modalities. However, he presented a significant diagnostic and medical decision‐making challenge when he repeatedly and temporally presented with what appeared to be a new‐onset CNS demyelinating condition without an obvious explanation other than the revumenib drug effect. On autopsy, these symptoms and radiologic signs surprisingly proved to be neither a demyelinating condition, nor a toxic revumenib (or other drug) effect, but rather, the evolution of uncontrolled leukemia localized to his CNS and PNS in a rare phenomenon known as neuroleukemiosis. In retrospect, it is likely that his progressive and eventually debilitating stocking‐glove mixed sensory/motor neuropathies, weakness, and pain were early signs and symptoms of neuroleukemiosis.

Neuroleukemiosis describes leukemic infiltration of PNs, resulting in axonal nerve injury. This is an exceedingly rare complication of leukemia with only a few published case reports [21]. Patients typically present with symptoms of a mononeuropathy, although asymmetric—and less commonly symmetric—multiple nerve involvement syndromes have also been reported [22]. The main differential diagnoses are GBS, Charcot–Marie–Tooth disease, treatment‐related toxicities, and abscesses. The gold standard for neuroleukemiosis diagnosis is PN biopsy. However, nerve biopsies are often not pursued due to both the low diagnostic yield from the sample collected and the risk of procedure‐associated complications such as infection, worsening of neuropathic symptoms, and significant pain and discomfort [23]. Patients receiving chemotherapeutic agents well known to cause a toxic neuropathy would not typically have a biopsy performed. However, for this patient, the rarity of a toxic neuropathy with his specific chemotherapeutic agents could raise consideration for a biopsy to help resolve the differential diagnostic considerations of an acquired inflammatory neuropathy, hereditary neuropathy, toxic neuropathy, or leukemic infiltration of the nerve. EMG testing can aid in the diagnosis of neuroleukemiosis, for which it will show decreased amplitude indicative of an axonal injury, as was found in our patient during his pre‐revumenib neuropathy work‐up.

As there is a blood–nerve barrier (BNB) analogous to the blood–brain barrier (BBB), the PNs serve as a chemotherapy‐protected sanctuary site not shared by the CNS, making neuroleukemiosis in the absence of CNS leukemia common [22]. Case reports of agents known to cross the BBB have been noted effective against neuroleukemiosis, including HD‐AraC, fludarabine, methotrexate, cyclophosphamide, etoposide, mitoxantrone, and radiation. Our patient had been exposed to many, but not all, of these agents. If a diagnosis of neuroleukemiosis had been made, by PN biopsy, for example, additional barrier‐penetrating agents could have been tried, including more extensive radiation to the brain and a modality to include the spine. Certainly, this evidence would have compelled us not to discontinue revumenib, although further study into this agent's ability to penetrate the BBB and BNB needs to be pursued. Overall, this case highlights a presentation of neuroleukemiosis, which should be included in the diagnostic schema of peripheral neuropathy in the setting of R/R AML.

An additional important reminder from this case is how difficult it is to assess toxicity attribution to drugs in general, but particularly in relatively novel drugs and ones used in ill patients. In this case, the patient's peripheral neuropathy was not because of presumed chemotherapy toxicity or chronic illness, and the patient's emergent radiologic findings post‐revumenib were not, as presumed, drug‐related. Toxicity attribution on clinical studies is notoriously inaccurate [24] and the same could be presumed clinically. Best practices, such as interrogating Bradford Hill criteria, need to be vigilantly utilized and ultimately improved upon when attempting to make clinical decisions about the effect of an agent [25].

## Ethics Statement

The legally authorized representatives (parents) provided written permission for the generation of this manuscript. Institutional review board approval is not required at our institution for the analysis or publication of single‐patient case reports.

## Conflicts of Interest

The authors declare no conflicts of interest.

## Data Availability

All data analyzed or generated during this study are included in the published manuscript. However, all quires or requests for clarification will be addressed by the corresponding author upon reasonable request.
